# Development of a SYBR green real-time PCR method for rapid detection of sheep pox virus

**DOI:** 10.1186/1743-422X-9-291

**Published:** 2012-11-27

**Authors:** Hong Tian, Jingyan Wu, Yan Chen, Keshan Zhang, Youjun Shang, Xiangtao Liu

**Affiliations:** 1 State Key Laboratory of Veterinary Etiological Biology, Lanzhou, 730046, China; 2 National Foot and Mouth Disease Reference Laboratory, Lanzhou, 730046, China; 3 Lanzhou Veterinary Research Institute, Yanchangbu, Xujiaping, Lanzhou, 730046, China; 4 Chinese Academy of Agricultural Sciences, Lanzhou, 730046, China

**Keywords:** Sheep pox virus, SYBR Green I based quantitative PCR

## Abstract

**Background:**

In this study, we developed a SYBR Green-based real-time PCR assay for the detection of sheep pox virus using a plasmid construct carrying one of the highly conserved genes encoding the virion envelope protein (P32) as a template.

**Results:**

The method was demonstrated to be highly sensitive, allowing a precise SPV DNA quantitation over a range of nine orders of magnitude (from 10^1^ to 10^9^ copies of standard DNA). Then, specimens from SPV suspected sheep were analyzed by conventional gel-based PCR, real-time PCR and sequence analysis.

**Conclusion:**

Comparison between these different techniques revealed that real-time PCR is more sensitive than conventional gel-based PCR, allowing detection low viral titers of SPV in infected sheep.

## Background

Sheep pox (SP) is malignant diseases of small ruminants causing heavy economic loss in the endemic countries [1,2]. The causative agents, sheep pox and goat pox viruses, belong to the genus *Capripoxvirus* in the family *Poxviridae*[3,4]. The diseases are endemic in India, Bangladesh, throughout the near and middle east, northern and central Africa [5].

Prompt detection of sheep pox virus (SPV) in the field samples is important for effective SPV control, thereby reducing the potentially serious economic damage which can result from an outbreak [6]. Because of the isolation of virus in cell cultures is technically difficult and time-consuming, so it is not a suitable routine diagnostic tool. Therefore, A rapid, specific and sensitive assays are required for the diagnosis of SPV. The aims of this study were to develop a rapid and sensitive method, able to detect a wide range of field samples of SPV in a feasible way in a short time.

## Results and discussion

SYBR green real-time PCR amplification was carried out with Mx3005P Real-Time PCR System (Agilent Stratagene, USA) and the data were analyzed with MxPro^TM^ QPCR System (version 4.10). Duplicates of the SPV standard dilutions and DNA templates were simultaneously subjected to real-time analysis. After 40 amplification cycles, a melting analysis was carried out to verify the correct product by its specific melting temperature (Tm). The optimal annealing temperature was 57 °C, whereas optimal primer concentrations were 0.4μM. The results were analyzed using MxPro^TM^ QPCR Software and Tm values were taken to verify the specificities of the PCR products.

**Figure 1:**
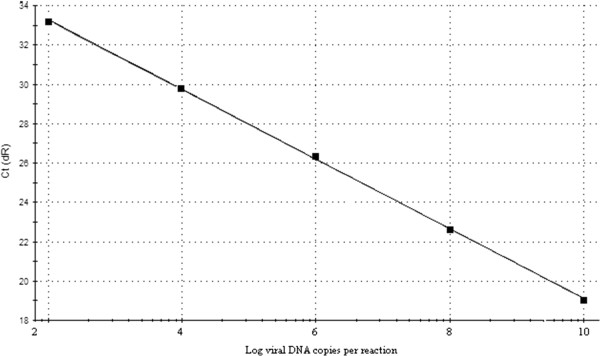
**Standard curve (plot of the *C***_**T**_**values against the different P32 plasmid DNA concentrations), showing a linear relationship between plasmid concentration and threshold cycles.** The standard formula is y = −3.528x + 15.60 and the correlation co-efficient is 0.999.

**Figure 2:**
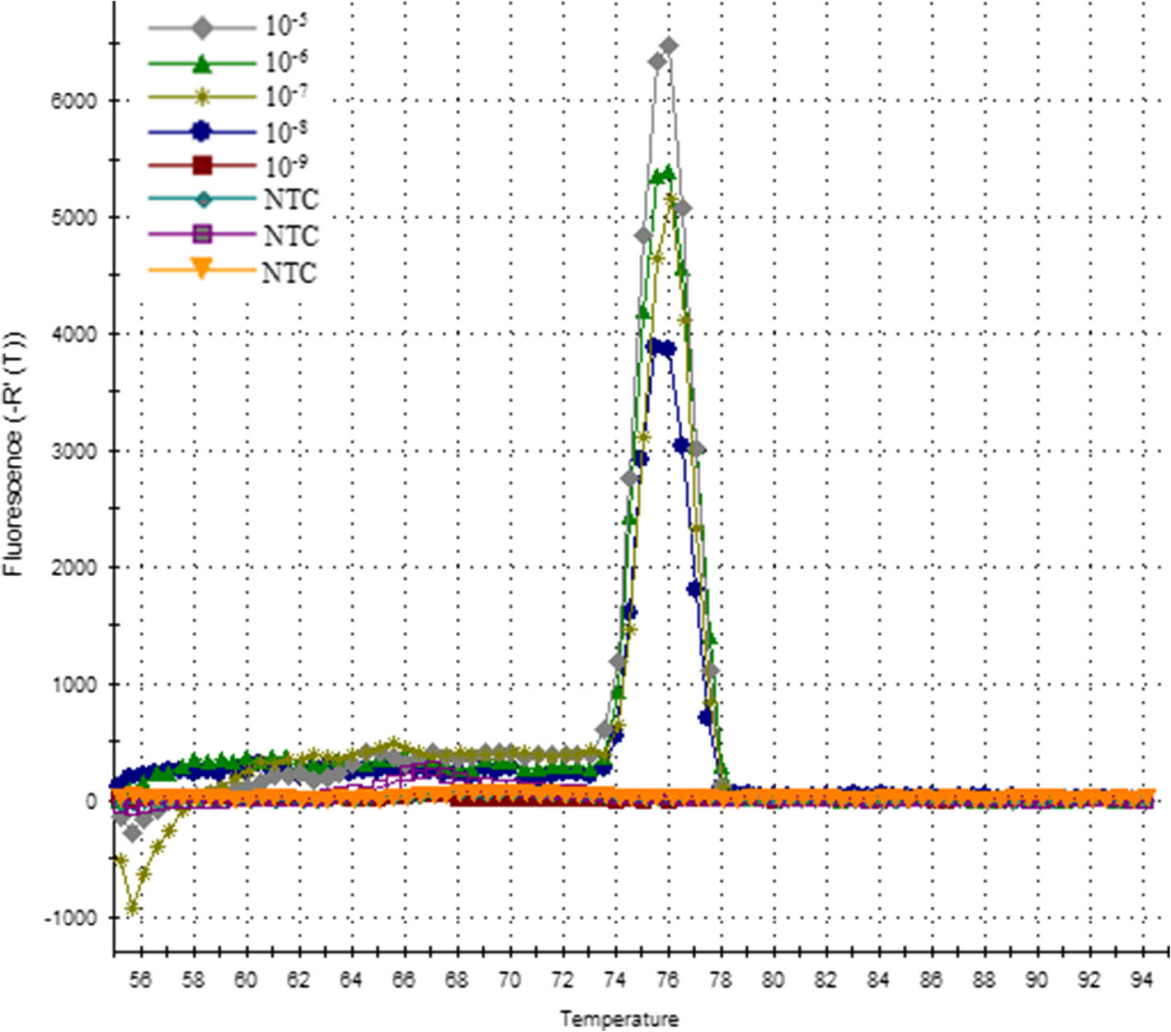
**The dissociation plot of amplified plasmid P32 DNA.** Melting peaks of P32 plasmid DNA ten-fold serial dilutions and negative control, the positive samples showed an identical melting curve profile.

In order to evaluate the reproducibility, a dilution end-point standard curve was made and repeated for three times. Ct values were measured in triplicate and plotted against the amount of plasmid copy number (Figure 1). The standard formula was y = −3.528x + 15.60 and the correlation co-efficient was 0.999. The diluted plasmid pMD-P32 was positive for a 10–8 dilution (Ct = 38.65), indicating a sensitivity of the method being 10 copy numbers per reaction mixture. No primer–dimers or non-specific amplification product were visible for negative samples (Figure 2).

To assess the use of the real-time PCR for the detection of viral DNA in the clinical samples. Seven of these 26 samples were found to be positive by conventional PCR, ten of 26 samples were found to be positive by real-time PCR. All samples identified positive by conventional PCR and real-time PCR were confirmed to be SPV by sequence analysis.

Real-time PCR assays have been widely utilized for early diagnosis of many other animal viral diseases [7,8]. In this study a real-time PCR assay was developed and evaluated for detection of SPV in field samples. The assay described in this report generates complete result in 1.5 h and can be used as a rapid diagnostic tool.

To improve the sensitivity of the method described it was necessary to optimize the conditions of primers and annealing temperature. With these parameters, the detection of the plasmid P32 could be up to a 10^−8^ dilution. This method does not require post-PCR manipulation because the melt curve data allow to verifying amplification products, thus diminishing the potential contamination risk. No primer–dimers were observed in the amplification products when analysed by melting curve. Under the above conditions, it was possible to establish a sensitivity of 10 copy numbers per reaction mixture.

## Conclusion

Considering the prevalence and economic impact of SPV, a simple, cost-effective, sensitive and rapid diagnostic technique is very important. The SYBR green real-time PCR assay described in this study has all these attributes. This technique has applications in routine diagnostics in common laboratories.

## Materials and methods

In this study, 26 field samples (scars) were collected from SPV suspected animals during 2009 in China during general surveillance. Samples were placed at −70°C for further use. The DNA of all field samples was extracted using QIAamp DNA Mini Kit (Cat. No.51306, Qiagen).

The primers used for real-time PCR amplification of SPV were designed using sequence data from the SPV P32 protein gene, and the primer will dilution into different concentration (final concentration, 0.2, 0.4, 0.6, or 0.8 μM). The partial sequence of the P32 gene of SPV was downloaded from GenBank (accession no. AY159333) and aligned (using Clustal W program in the MegAlign Package (DNAStar)) with the available P32 gene sequences of other strains of SPV to identify the conserved regions. Primers were designed and synthesized target on conserved regions (Table 1). The routine PCR reaction mixture containing the amplified 900 bp products was cloned into the pMD18-T Vector (TaKaRa Code, D101A). The plasmid P32 DNA was used as PCR standard template for the determination of the standard curve and sensitivity of the real-time PCR. After DNA quantitation by NanoDrop ND-2000 analysis, 10-fold dilutions of the plasmid, representing10^0^-10^9^ copies of DNA/10 μL of template, were used to carrying out the standard curve and the sensitivity of developed real-time PCR methods. Each dilution were frozen at −70°C and used only once.

**Table 1:** Oligonucleotide primers designed for SPV amplification by conventional gel-based PCR and real-time PCR

**Primer**	**Sequence (5**^′^**-3**^′^**)**	**Length**	
		**Primer (bp)**	**Product (bp)**
Nf	GGGGGATATGATTTTACCTTA	21	235^a^
Nr	ATATACCGTTTTTCATTTCGTTAG	24	
900f	TTATATGTTATACCAATCGTTGGTC	25	900^b^
900r	TAACATACCTGCTAAAAACCAT	22	

^a^ Length of product amplified by real-time PCR primer pair.

^b^ Length of product amplified by conventional PCR primer pair.

## Competing interests

None of the authors of this paper has a financial or personal relationship with other people or organizations that could inappropriately influence or bias the content of the paper (including National Modern Meat Caprine Industrial Technology System).

## Authors’ contributions

HT participated in design experiments and drafted the manuscript. JYW and YC carried out the experiments. KSZ and YJS conceived of the study. XTL participated in its design and coordination. All authors read and approved the final manuscript.
